# Inhibition of complement improves graft outcome in a pig model of kidney autotransplantation

**DOI:** 10.1186/s12967-016-1013-7

**Published:** 2016-09-23

**Authors:** Pierre-Olivier Delpech, Raphael Thuillier, Thibault SaintYves, Jerome Danion, Sylvain Le Pape, Edwin S. van Amersfoort, Beatrijs Oortwijn, Gilles Blancho, Thierry Hauet

**Affiliations:** 1 Département d’Urologie, CHU de Poitiers, 86000 Poitiers, France; 2 Inserm U1082, 86000 Poitiers, France; 3 Service de Biochimie, CHU Poitiers, 86000 Poitiers, France; 4 Faculté de Médecine et de Pharmacie, Université de Poitiers, 86000 Poitiers, France; 5 Fédération Hospitalo-Universitaire SUPORT, 86000 Poitiers, France; 6 Département D’urologie, CH D’Angoulème, 16000 Angoulème, France; 7 Service de Chirurgie Viscérale, CHU de Poitiers, 86000 Poitiers, France; 8 Pharming Technologies BV, NL-2333CR Leiden, The Netherlands; 9 Institut de Transplantation Urologie et Néphrologie (ITUN), CHU de Nantes, Faculté de Médecine et des Techniques Médicales de Nantes, Université de Nantes, Inserm U1064, 44000 Nantes, France; 10 Institut National de La Recherche Agronomique, Unité Expérimentale Génétique, Expérimentations et Systèmes Innovants, Domaine Expérimental Du Magneraud, Plateforme IBiSA ‘MOPICT’, 17700 Surgères, France; 11 INSERM U1082, CHU de Poitiers, 2 Rue de La Miletrie, 86021 Poitiers Cedex, France

**Keywords:** Complement system proteins, Drug evaluation, Ischemia, Kidney transplantation, Preclinical, Reperfusion injury

## Abstract

**Background:**

Ischemia reperfusion injury (IRI) induced immune response is a critical issue in transplantation. Complement and contact system activation are among its key mechanisms.

**Study design:**

We investigated the benefits of pre-reperfusion treatment with recombinant human C1INH (rhC1INH), inhibitor of both complement and contact activation, in a pig model of kidney autotransplantation, subjecting the organ to 60 min warm ischemia prior to 24 h static preservation to maximize damage.

**Results:**

Serum creatinine measurement showed that treated animals recovered glomerular function quicker than the Vehicle group. However, no difference was observed in tubular function recovery, and elevated level of urinary NGal (Neutrophil gelatinase-associated lipocalin) and plasma AST (Aspartate Aminotransferase) were detected, indicating that treatment did not influence IRI-mediated tubular cell necrosis. Regarding chronic graft outcome, rhC1INH significantly prevented fibrosis development and improved function. Immunohistochemistry and western blot showed decreased invasion by macrophages and T lymphocytes, and reduction of epithelial to mesenchymal transition. We determined the effect of treatment on complement activation with immunofluorescence analyses at 30 min post reperfusion, showing an inhibition of C4d deposition and MBL staining in treated animals.

**Conclusions:**

In this model, the inhibition of complement activation by rhC1INH at reperfusion, while not completely counteracting IRI, limited immune system activation, significantly improving graft outcome on the short and long term.

**Electronic supplementary material:**

The online version of this article (doi:10.1186/s12967-016-1013-7) contains supplementary material, which is available to authorized users.

## Background

The rise of transplantation science to the status of the most adapted treatment for end-stage renal disease increased the demand for organs. As a consequence, only one quarter of patients on waiting lists have access to an organ. This led to the extension of donor criteria and the acceptation of marginal donors [1], donors aged over 60, and donors aged 50–59 with at least two of three additional risk factors including cerebrovascular accident as a cause of death, history of hypertension, and serum creatinine above 1.5 mg/dl prior to transplantation. However, these organs are particularly sensitive to ischemia reperfusion injury (IRI) [2], well demonstrated as having a dramatic impact on short [3] and long term [4] outcome. Current organ preservation techniques are not adapted to these new donor demographics, and it is thus of primary importance to better define IRI mechanisms in order to devise novel therapeutic protocols and improve organ quality.

The complement cascade is a key feature of the immune response, capable of inducing both innate and adaptive responses as well as lead to cell death [5]. In recent years, evidence has accumulated towards the involvement of this pathway in transplantation complications. The complement system is activated immediately following reperfusion, continuing through the multiple stages of graft survival [6], from the early response to chronic fibrosis development [7]. Indeed, complement is already involved at the donor level, playing for instance a key role in the tissue damage occurring during brain death [8].

The involvement of complement in IRI has been extensively demonstrated in a variety of mouse models [9], using multiple KO approaches for key factors such as C3, Factor B, C5, or DAF. This prompted the testing of complement-targeted therapies against IRI [10]: in rats, an analog of C3 convertase inhibitor protected against ischemia reperfusion (IR) complications; in mice, a Mannose-Binding Lectin (MBL) inhibitor prevented thrombogenesis, while monoclonal antibodies against MBL reduced complement deposition in vitro and post-myocardial infarction lesion in rats; recombinant soluble complement receptor 1 was beneficial in models of IR and in models of transplantation (lung and liver). The importance of complement activation in reperfusion injury prompted the initiation of clinical trials to test the benefits of an anti-C5 antibody (Eculizumab) to prevent DGF (Delayed Graft Function) (NCT01403389; NCT01919346), which are still ongoing.

In the current study, we tested the benefits of a recombinant human C1 inhibitor (rhC1INH) [11]. Therapeutic strategies centered on C1 have shown significant benefits in models of myocardial IR [12] as well as in lung transplantation models [13]. This strategy downregulated the expression of surface receptors on endothelial cells, reducing the immune response [14]. Endogenous C1 inhibitor (C1-INH) has a plasma concentration ∼240 mg/l, corresponding to 1 U/ml [15]. It belongs to the serpin family (serine protease inhibitors) and while it targets the classical pathway, recombinant C1 inhibitors have been shown to target the lectin pathway as well in myocardial IR models [16], and to have several inhibitory activities on the alternative pathway, particularly through C3b [17].

C1INH also exhibits activities beyond its original target C1, as indeed it was demonstrated to target factor XIIa and plasma kallikrein of the contact system, factor XIa and thrombin of the coagulation pathway as well as plasmin and tissue plasminogen activator of the fibrinolytic pathway [18]. Thus, this compound could have protective benefits beyond its actions on the complement pathway. Indeed, our own work [19, 20] highlighted the therapeutic benefits of using a molecule directed against the coagulation pathway in a preclinical model of kidney transplantation.

RhC1INH inhibited complement deposition in a large animal model of kidney warm ischemia [21]. This prompted us to test rhC1INH in a preclinical model of kidney autotransplantation in the pig, an animal offering a very high degree of correlation to the clinical situation [22], in which the kidney was subjected to a period of 60 min warm ischemia prior to retrieval in order to maximize IRI. The experiments in this report demonstrate that a single dose of rhC1INH at the time of reperfusion reduces complement deposition and improves long term function of the transplanted kidney.

## Methods

### Compound

Both recombinant C1 esterase inhibitor (rhC1INH; Ruconest^®^) and vehicle were provided by Pharming B.V. (Leiden, The Netherlands) rhC1INH concentration was 150 U/mL. Vehicle contained sodium citrate (19.7 mol/L), citric acid (0.3 mol/L), sucrose (189.9 mol/L); pH 6.8.

### Animal model

Surgical and experimental protocols were performed in accordance with French Ministry of Agriculture, National Institute for Agronomic Research and Poitou–Charentes ethical comity of animal experimentation (protocol number CE2012-4). We used 3 months old Large White pigs (40 ± 4 kg, IBiSA, INRA Magneraud, France).

As previously described [23], after anesthesia the left kidney was approached through a midline abdominal incision. The left renal vascular pedicle and ureter were dissected, and warm ischemia induced by 60 min renal pedicle clamping. Unfractionated heparin (UFH, 125 UI/kg) was injected 10 min before clamping. The kidney was then removed and flushed with 4 °C University of Wisconsin preservation solution (UW) supplemented with 5000 IU/L UFH and stored at 4 °C for 24 h.

After preservation, the kidney was transplanted into the same animal by heterotopic autotransplantation. End-to-side aorta and inferior vena cava anastomoses and ureteroneocystostomy were performed.

The half-life of RhC1INH in patients is approximately 3 h for a 100 U/kg injection [24, 25]. Considering that the drug was well tolerated and that a previous study showed observable effects in the pig [21] for a 500 U/kg injection, we decided to inject RhC1INH (500 U/kg) or vehicle via the central line 15 min before unclamping. This timing is consistent with the distribution of the compound in both animals and humans, showing that it reached an effective dosage within 15–30 min [24]. The contralateral kidney was removed 25 min after unclamping and biopsied to measure the compound effect on a non-ischemic organ. The transplanted kidney was biopsied 30 min after unclamping.

Two groups were studied: Vehicle (VEH): injected with 3.33 mL/kg vehicle 15 min before unclamping, rhC1INH: Injected with 3.33 mL/kg rhC1INH (500 U/kg) 15 min before unclamping. In case baseline values were needed, measurements or staining where performed in healthy littermates (Controls, CTL: values are indicated for reference and not included in the statistical analysis. 7 animals were used for each group, as determined by power calculations (Additional file 1: Tables S1, S2).

### Renal function evaluation

Plasma creatinine (modified compensated Jaffé technique), plasma aspartate aminotransferase (AST) and alanine aminotransferase (ALT) (IFCC procedure with pyridoxal phosphate) were measured with a COBAS bioanalyser (Roche-Diagnostics, France).

### Urinary Ngal quantification

Urine levels of Ngal (Neutrophil gelatinase-associated lipocalin) were measured by ELISA (Eurobio, France) following the manufacturer’s recommendations.

### Morphological study

Immunofluorescence: biopsy samples of kidney cortex were processed for cryosectioning. Antibodies: Anti Factor B (Novus Biologicals, UK), anti C4d, anti C1q (AbD Serotec, France), anti C3c, anti C5b-9 (DAKO, France), anti MBL, anti MASP2 (Cliniscience, France). Each slide was semi quantitatively evaluated by a trained nephropathologist.

Immunohistochemistry: biopsies were preserved in formalin. Antibodies: anti-vimentin (Cell-Marque, USA), anti-αSMA (alpha smooth muscle actin); (Sigma, St. Louis, MO), anti-Calprotectin reacting with Monocyte/Granulocytes/Macrophages (MAC-387, AbCam, France) and anti-CD3 (SouthernBiotech, Birmingham, Alabama, USA). Fibrosis was analyzed by Sirius Red staining. Staining quantification was performed in silico (Visilog 6.9 software): fibrosis: percent of stained area per field; inflammation: number of positive immune cells *per* field. We evaluated 10 fields (×100) per tissue sample.

### Western blot procedure

We used specific antibodies against Transforming Growth Factor-β, Phospho Smad 3, Smad 3 and BMP7 (Santa-Cruz, France) and β actin as loading control (A1978; Sigma Aldrich, France).

#### Statistics methods

We used NCSS software (NCSS LLC, USA). Normality was tested using the Skewness, Kurtosis and Omnibus tests while equality of variance was asserted using the modified-Levene Equal-Variance. In case these parameters were met, a two-sample *T* test analysis was performed, otherwise a Mann–Whitney U test was used. Data sets were tested for the presence of outliers using Grubbs’ test. Statistical power calculations were performed using Anastat (http://www.anastats.fr/). Statistical significance was accepted for p < 0.05.

## Results

### Early outcome

**Fig. 1:**
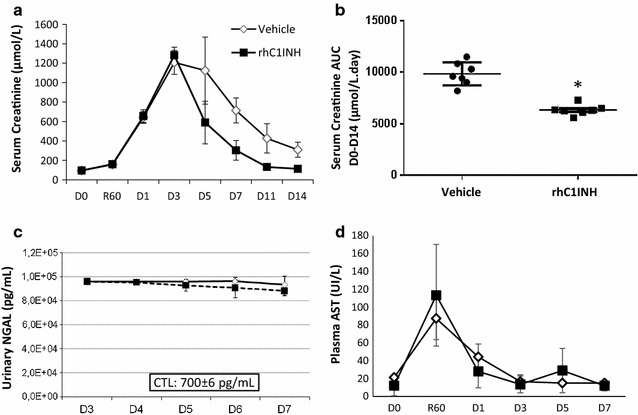
Early outcome: function and tubular necrosis. **a**, **b** Evolution of serum creatinine in the first 2 weeks post-transplant. Serial blood samples were collected from transplanted pigs and processed for biochemical analysis. **a** serum creatinine levels profiles in both groups. **b** Area under the curve comparison between the two groups. **c** Urinary Ngal levels during the first week post reperfusion. Serial samples were collected and levels of Ngal evaluated with an ELISA technique. Reference value for CTL is CTL: 700 ± 6 pg/mL. **d** Plasma levels of aspartate aminotransferase (AST) evaluated by IFCC procedure with pyridoxal phosphate. Shown are mean ± SD, n = 7. Statistics: *p < 0.05. AUC data was normally distributed and had equal variance, hence parametric analysis was performed

Serial measurements (Fig. 1a) showed that the VEH treated animals had a rapid rise in serum creatinine until day 3, with a peak at 1200 µmol/L. Levels remained high until day 5 then slowly decreased, reaching ~300 µmol/L at day 14, more than three times the pretransplant levels. In the rhC1INH group, time to peak and height of the peak were similar to that of VEH animals; however serum creatinine levels started to decrease immediately (day 1) to reach ~300 µmol/L by day 7, and ~100 µmol/L at day 14, not significantly different from pretransplant levels. Area under the curve comparison showed that rhC1INH treatment significantly improved kidney function recovery (p = 0.002, Fig. 1b).

Fraction of excreted sodium analysis (Additional file 2: Fig. 1a), showed no difference between groups, with elevated sodium fraction at D3 recovering to pretransplant levels by D7. Tubular injury marker urinary Neutrophil gelatinase-associated lipocalin (Ngal) levels were high at day 3, with ~10,000 pg/mL in both groups, while the baseline in Large White pigs is ~700 pg/mL. These remained high until D7 (Fig. 1c). We also investigated plasma AST (Fig. 1d), which was strongly increased immediately after reperfusion, with a peak at 60 min ~110 UI/L. This was followed by a decrease to pretransplant levels by day 3. There was no difference between groups, suggesting that rhC1INH does not directly affect tubular IR lesions (Additional file 2: Fig. 1).

### Chronic outcome: function

**Fig. 2:**
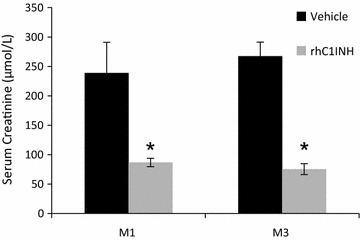
Chronic function. Evolution of serum creatinine at months 1 and 3 of post-transplant follow up. Shown are mean ± SEM, n = 7. Statistics: *p < 0.05. Serum creatinine values at M1 and M3 were normally distributed but did not show equality of variance on the Modified-Levene Equal-Variance Test, hence non parametric analysis was performed

Serum creatinine measurements during the 3 months follow up showed that function remained poor in the VEH group, with levels ~250 µmol/L, close to threefold the baseline (95 µmol/L, Fig. 2). On the other hand, levels in the rhC1INH group further decreased at month 1 to ~80 µmol/L (from 113 µmol/L at day 14), not different from pretransplant measurements (p = 0.002, VEH vs. rhC1INH at 3 months).

### Chronic outcome: interstitial fibrosis

**Fig. 3:**
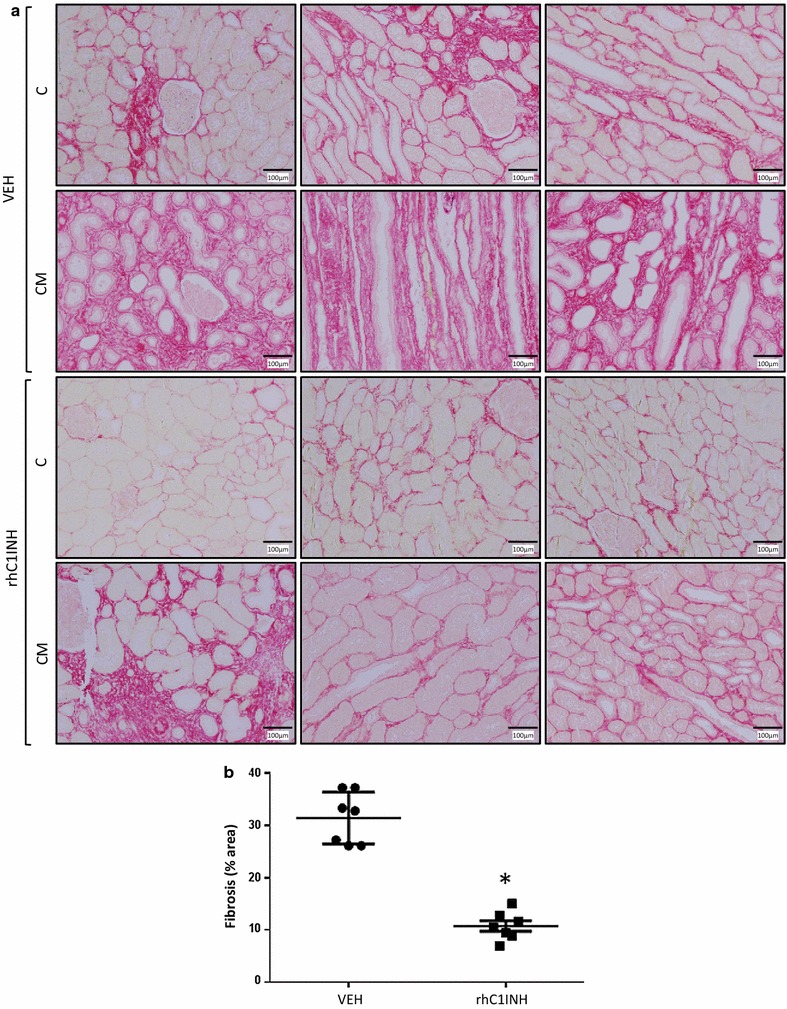
Chronic fibrosis development at 3 months follow up. Pigs were euthanized after 3 months follow up and kidney biopsies were collected and processed for immunohistochemical analysis. Sirius Red labelling and quantification was performed. **a** Representative staining for each group in the cortex (C) and corticomedullar (CM) areas. **b** quantification of the area covered by the staining per high powered field (×100). Shown are mean ± SD, n = 7. Statistics: *p < 0.05. Fibrosis data were normally distributed and had equal variance, hence parametric analysis was performed

To better explain the functional phenotype, we determined the emblematic lesions of chronic failure: interstitial fibrosis and tubular atrophy (IFTA). Three months after transplantation, the animals were euthanized and kidney samples from cortical and corticomedullary areas were subjected to Sirius Red staining (Fig. 3). There was a clear development of IFTA in the VEH group, ~30 % of the observed area, demonstrating substantial chronic injury. On the other hand, fibrosis in the rhC1INH group was limited to a third of that observed in the VEH group (~10 %, p = 0.002 to VEH).

### Chronic outcome: innate immunity and epithelial to mesenchymal transition

**Fig. 4:**
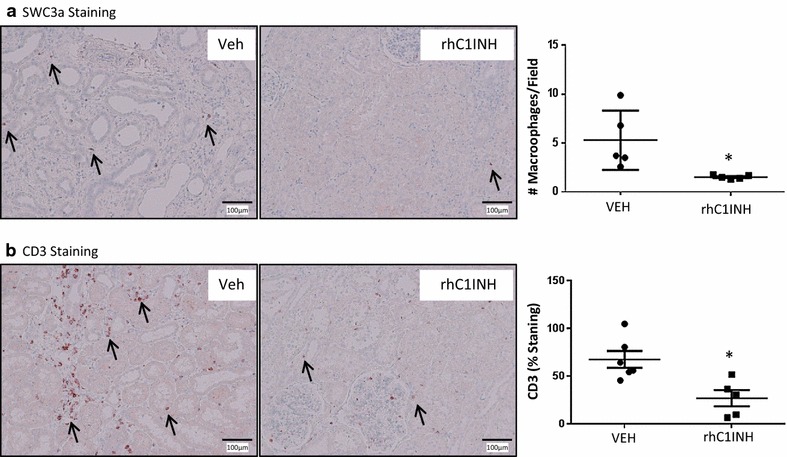
Immunohistochemical staining on 3 months post-transplant kidney samples: Immune cell infiltration. Pigs were euthanized after 3 months follow up and kidney biopsies were collected and processed for immunohistochemical analysis. **a** MAC-387 staining, detecting macrophages in the pig. *Left* and *middle* representative staining for each group. Arrows designate positive cells. Magnification: ×100. *Right* quantification of the number of positive cells per high powered field (×100). **b** CD3 staining, specific for T lymphocytes. *Left* and *middle* representative staining for each group. *Arrows* designate positive cells. Magnification: ×100. *Right* quantification of the number of positive cells *per* high powered field (×100). Shown are mean ± SD, n = 7. Statistics: *p < 0.05. MAC-387 data were normally distributed but did not show equality of variance on the Variance-Ratio Equal-Variance Test, hence non parametric analysis was performed. CD3 data was normally distributed and had equal variance, hence parametric analysis was performed

To determine the possible source of IFTA development, we investigated innate and adaptive immune cells population within the graft at 3 months using immunohistochemistry (Fig. 4). MAC-387 staining (Fig. 4a) revealed the presence of macrophages within the thickened interstitium, while the number of cells within the field of the rhC1INH treated kidneys remained significantly low (p = 0.009), suggesting that the treatment limited the development of innate immune response within the graft. We further investigated tissue invasion by T lymphocytes (Fig. 4b). CD3 staining revealed an important invasion in the VEH group (approx. 65 cells/field), which was significantly reduced in the rhC1INH group (approx. 25 cells/field, p = 0.011). This suggests that the treatment protected the kidney against the T lymphocyte arm of the chronic immune response.

**Fig. 5:**
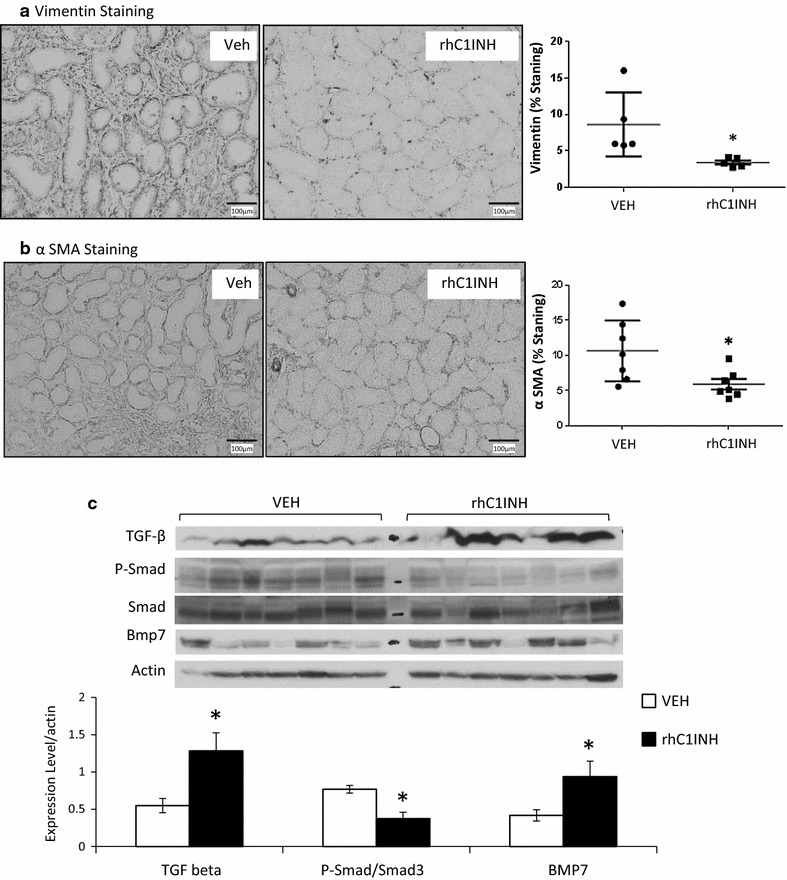
Immunohistochemical staining on 3 months post-transplant kidney samples. Pigs were euthanized after 3 months follow up and kidney biopsies were collected and processed for immunohistochemical analysis. **a** Vimentin staining, specific for EMT. *Left* and *middle* representative staining for each group. Magnification: ×100. *Right* quantification of the area covered by the staining per high powered field (×100). **b** α-SMA staining, specific for EMT. *Left* and *middle* representative staining for each group. Magnification: ×100. *Right* quantification of the area covered by the staining per high powered field (×100). **c** Western blot analysis of TGF-β signaling; top: representative blots for each marker; bottom: quantification of the signal normalized to β-actin. Shown are mean ± SD, n = 7. Statistics: *p < 0.05. Vimentin staining data were normally distributed but did not show equality of variance on the Variance-Ratio Equal-Variance Test, hence non parametric analysis was performed. α-SMA staining data were normally distributed and had equal variance, hence parametric analysis was performed. TGF-β protein level values were normally distributed but did not show equality of variance on the Variance-Ratio Equal-Variance Test, hence non parametric analysis was performed. P-Smad3/Smad3 ratio values were normally distributed and had equal variance, hence parametric analysis was performed

We then investigated epithelial to mesenchymal transition (EMT) using vimentin and αSMA as markers of differentiating cells (Fig. 5a, b). In VEH animals, staining was observed inside the tubules as well as in the interstitium, indicating the presence of tubular cells acquiring characteristics of mesenchymal cells followed by migration outside of the tubules. On the other hand, staining in the rhC1INH group was more localized, involving a reduced number of tubular and interstitial cells (p = 0.009 to VEH). Furthermore, αSMA staining showed significantly higher levels in the interstitium of VEH kidneys compared to rhC1INH organs in which staining was more limited (p = 0.018 to VEH). This is in accordance with IFTA results showing a reduction of injury development in the treated group suggesting a potential protection against collagen deposition and tubular atrophy.

We thus explored upstream signaling of EMT, using Western blotting to detect markers of the TGF-β pathway (Fig. 5c). We determined that whereas TGF-β protein was higher in the rhC1INH group than in the VEH kidneys, the activation level of Smad 3 was higher in the VEH groups. This correlated with BMP-7 levels, a TGF-β antagonist, which remained high in the rhC1INH group. This discrepancy between TGF-β production on the one hand and Smad3/BMP7 on the other could suggest a role for TGF-β not related to fibrosis development as suggested by the red Sirius staining.

### Complement deposition 30 min post reperfusion

Immunofluorescent staining was performed on 30 min biopsies to determine the extent of complement deposition.

**Fig. 6:**
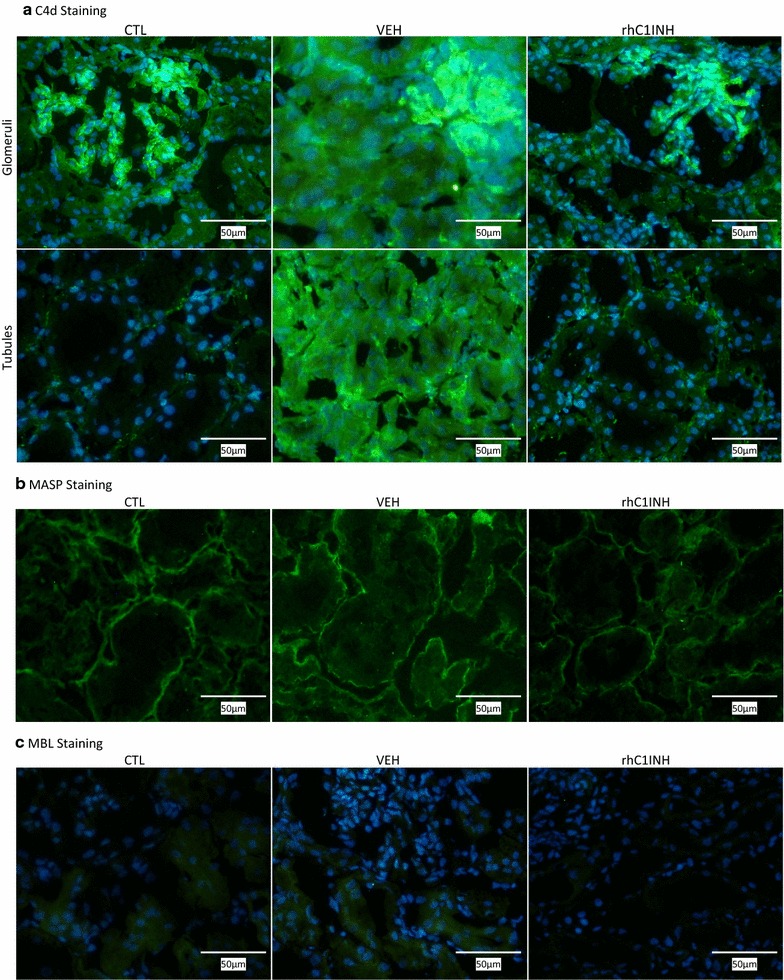
Immunofluorescent evaluation of complement deposition in kidney biopsies at 30 min post-transplant: C4d, MASP and MBL. Needle biopsies were performed 30 min after unclamping (45 min post injection) and processed for cryopreservation. Slides were evaluated by fluorescent microscopy by a trained nephropathologist. CTL: Healthy kidney; Vehicle (VEH): injected with vehicle 15 min before unclamping; rhC1INH: Injected with 500 U/kg rhC1INH 15 min before unclamping (n = 7). **a** Representative staining for C4d; *top row* glomerular staining; *bottom row* tubular staining. **b** Representative staining for MASP. **c** Representative staining for MBL. Magnification: ×200

C4d staining in the CTL group was intense in the glomeruli and mild in the peritubular capillaries (Fig. 6a; Table 1). At 30 min after transplantation, C4d deposition was greatly increased in both locations, particularly at the level of the peritubular capillaries (VEH). Moreover, the VEH group displayed intense C4d staining in the tubules. In the rhC1INH treated group however, C4d deposition was similar to CTL.

**Table 1:** Semi-quantitative evaluation of complement staining 30 min post-reperfusion

		CTL	VEH	rhC1INH
C1q	Glomeruli	++	++	++
	Vessels	+	+	+
C3c	Mesengium	++	+++	+++
	Glomerular capillary wall	+++	+++	+++
	Apex proximal tubules	–	+++	+++
C4d	Glomeruli	+++	+++++	+++
	Peritubular capillaries	++	++++	++
MASP	Tubular basal membrane	++	++	++
MBL	Intratubular	+	+	±
C5b-9		No staining	No staining	No staining
Factor B		No staining	No staining	No staining

Needle biopsies were performed 30 min after unclamping (45 min post injection) and processed for cryopreservation. Slides were evaluated by fluorescence microscopy by a trained nephropathologist

Mannan-binding lectin serine protease 1 (MASP) was detected at the tubular basement membrane in CTL groups (Fig. 6b; Table 1). This pattern remained unchanged after transplantation in both treatment groups.

**Fig. 7:**
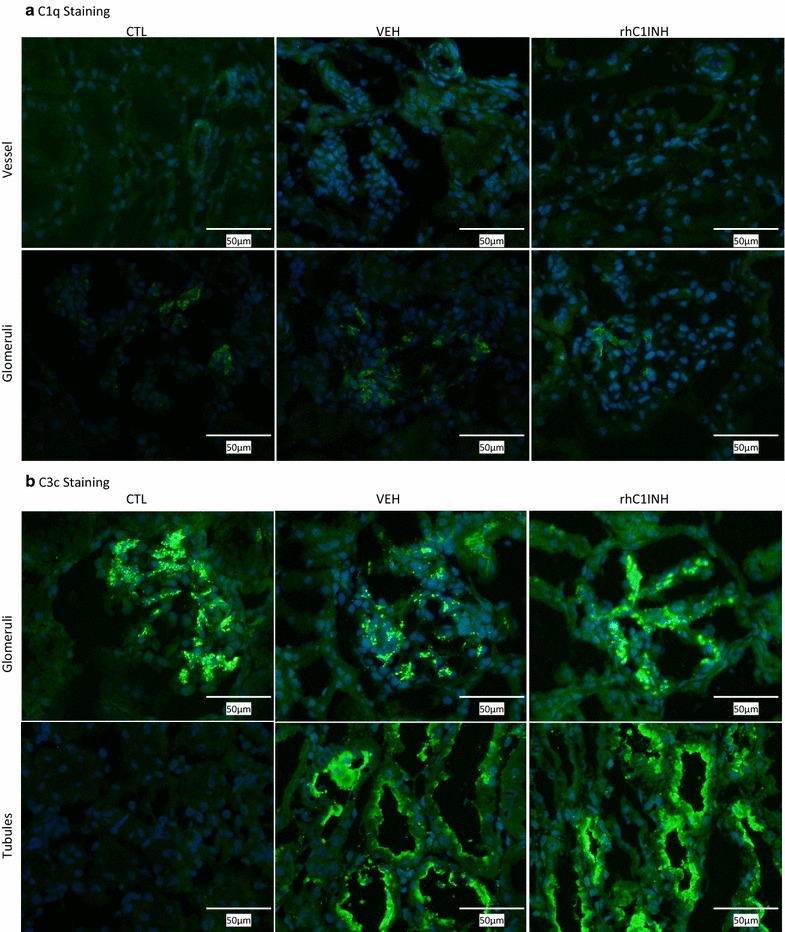
Immunofluorescent evaluation of complement deposition in kidney biopsies at 30 min post-transplant: C1q and C3c. Needle biopsies were performed 30 min after unclamping (45 min post injection) and processed for cryopreservation. Slides were evaluated by fluorescent microscopy by a trained nephropathologist. CTL: Healthy kidney; Vehicle (VEH): injected with vehicle 15 min before unclamping; rhC1INH: Injected with 500 U/kg rhC1INH 15 min before unclamping (n = 7). **a** Representative staining for C1q; *top row* vessel staining; *bottom row* glomerular staining. **b** Representative staining for C3c; *top row* glomerular staining; *bottom row* tubular staining. Magnification: ×200

Staining for C1q in healthy kidneys showed moderate glomerular staining as well as weak staining in the wall of small vessels (Fig. 7a; Table 1). IR did not alter this staining (VEH group) and the treatment with rhC1INH did not alter this pattern either.

In CTL, important basal C3c deposition was detected in the glomeruli (Fig. 7b; Table 1). Following transplantation, there was an important C3c deposition at the apex of proximal tubules (VEH group) and glomerular deposits were unchanged. Treatment with rhC1INH did not affect this staining pattern.

There was a weak Mannan-binding lectin (MBL) staining in the tubules in the CTL group (Fig. 6c; Table 1). Post-transplantation, this staining remained detected in the VEH kidneys, but it was weaker in kidneys of rhC1INH treated animals.

There was no detected staining for both C5-b9 and Factor B in any of the groups (Additional file 3: Fig. S2; Table 1).

## Discussion

In this study, we tested the relevance of targeting the complement system activation at the reperfusion stage in an auto-transplanted pig kidney model, using a compound with a wide range of activities, including inhibition of the complement, contact system, coagulation and fibrinolysis cascades.

RhC1INH treatment demonstrated a significant improvement of kidney function post-transplant. Measurement of serum creatinine levels showed that time to peak and height of peak were not altered, however there was a striking difference in recovery: while vehicle treated animals showed a slow recovery, rhC1INH treatment accelerated recovery with an earlier start (day 3) and sharper decreases reaching pretransplant levels within 14 days.

We investigated the chronic consequence of treatment: (i) function analysis showed that the rhC1INH- treated group recovered to pretransplant serum creatinine levels by month 1, while vehicle-treated animals never achieved levels below 250 µmol/L, threefold above baseline; (ii) IFTA exploration demonstrated that the treatment significantly reduced injury development; however rhC1INH-treated group levels were ~10 %, whereas in our experience with the same model, IFTA development could be reduced to 5 % [19]; (iii) immune response at 3 months was increased in the vehicle group, while it was lower in the rhC1INH-treated group; (iv) EMT activation was high in the vehicle group with both stainings observable in both the tubules and the interstitium of rhC1INH-treated kidneys.

Investigating further, we determined by western blot the activation of the signaling pathway emblematic of EMT and IFTA, i.e. TGF-β. This showed that rhC1INH treatment permitted some degree of protection, with reduced activation of Smad 3 and increased levels of BMP7. However, the observed increase in TGF-β expression in the rhC1INH group is surprising. This could be due to the fact that the treatment only partially protected against IRI. Our results are similar to another study on rhC1INH treatment and EMT [26], however the later uses a 30 min warm ischemia model in the pig, with outcomes measured after 24 h of reperfusion, hence representing a direct effect of the molecule, while herein we investigated the chronic consequences of the treatment. Hence, the treatment may have simply delayed the occurrence of fibrosis through the TGF-β pathway. Another explanation stems from the other role of TGF-β, namely as an anti-inflammatory cytokine [27, 28]. Indeed, the observed production of TGF-β could have a systemic effect, reducing immune activation. This hypothesis is in compatible with our results on innate and adaptive immune cell invasion.

We characterized the impact of the treatment on transplanted kidneys. We thus tested RhC1INH impact on complement activation using immunofluorescent staining on biopsies collected 30 min after reperfusion. In healthy pigs, complement pathway effectors are absent from the kidneys [21]. In our hands, rhC1INH noticeably inhibited C4d deposition. We did not see an effect of the treatment on C1q, C3c or MASP staining, but rhC1INH also appeared to affect MBL staining, reducing its signal. However this was detected inside the cells, an atypical, although described [29], localization for this protein, and too little data is available to permit definite conclusions. Thus, rhC1INH had some effect on complement deposition, mostly C4d. This is surprising, as C4 is upstream of C3 in the complement activation cascade; however, C4d deposition is more stable, and associated to pathways beyond the cytotoxic consequences of the complement cascade, such as antibody-mediated rejection [30]. RhC1INH was injected 45 min before the kidney was biopsied. Previous studies in humans and animals showed that it distributed rapidly and reached an effective dosage within 15–30 min [24], hence its high ratio of distribution and fast effect on complement could explain the lack of effects observed on its primary targets: the primary effect would have already taken place, and we only observe their consequences. Earlier biopsies could have revealed more, however the risk associated with the procedure was contrary to the aim of the study and ethical consideration.

Our results have commonalities with a recent study using rhC1INH in a pig warm ischemia model [21]. Indeed, comparing only animal data, both studies demonstrated a beneficial effect of the molecule on C4d deposition, and an influence of the treatment on the lectin pathway (MASP in the warm IR model, MBL herein). Other results in the warm IR model were not reproduced in our hands, such as the effect on C5b-9, however these differences can be explained by specificities of the models used (30 min warm ischemia versus 60 min warm ischemia combined to cold storage), and the timing of treatment, which are likely to induce different complement kinetics. Furthermore, as discussed above, the timing of our biopsies is perhaps not adapted to detect the early effects of the compound. Indeed, our setting could not permit us to perform serial biopsies on the organ, as it was required to sustain life for 3 months, hence we may have missed the activation of a specific pathway or its inhibition. All in all, both studies concurred to shown that rhC1INH was able to impede complement activation, and our results demonstrate the long lasting benefits of such a strategy on graft outcome.

Treatment at reperfusion involves a risk, and may be unable to fully prevent IRI. Indeed, this injury starts at the time of organ collection and extends well throughout the post-reperfusion period. Result herein show that the treatment did not prevent tubular injury, as shown by measurement of sodium fraction in the urine, as well as urinary Ngal and circulating AST detection, increasingly acknowledged as important non-specific markers of kidney lesion [31, 32]. Absence of difference between groups highlights the limits of treatment at reperfusion: while the benefits are evident in regards to glomerular function, tissue damage is taking place and may impact outcome. Our results suggest that treatment likely protects against the consequences of IRI on the inflammatory response, activated in autotransplanted kidneys, likely on the innate arm of the response. It would thus affect the ability of the kidney to recover from the injury, rather than the level of the injury itself, hence the absence of difference until day 3 (IRI effects at the tissue level) but increased recovery after day 3 (unimpeded repair). Thus, while rhC1INH treatment demonstrates evident benefits to graft outcome, combining this strategy with other methods directed at cold ischemia could be even more beneficial.

Ruconest has already been tested in clinical trials, concerning its safety (NCT00851409) and the treatment of Hereditary Angioedema (NCT00262301, [33]) and it is currently under further clinical evaluation for this pathology. Such an advanced clinical development is rarely found in transplantation-related IR treatments and thus represents an opportunity for a rapid deployment of complement-inhibition based therapy at the patient level.

## Conclusion

In conclusion, complement-directed therapy at reperfusion is beneficial to kidney grafts, improving function recovery and limiting chronic lesion development. However, this therapy may not be sufficient by itself to fully prevent IR-related damage, particularly EMT activation and IFTA development. In the current organ shortage, decreased donor organ quality requires new conceptual developments in organ preservation and emerging recipient care protocols. RhC1INH could be an integral part of such optimized protocols in conjunction with other agents, for instance targeting oxidative stress [4] and other key reperfusion pathways such as coagulation [19], or following machine perfusion [34] in order to obtain a maximal level of protection against IR, particularly in marginal donors situations.

## Additional files

10.1186/s12967-016-1013-7 Power calculations for the different statistical analyses. Calculations were performed using Anastat (http://www.anastats.fr/) with an alpha threashold of 0.05. **Table 2. ** Immune cell counts.
10.1186/s12967-016-1013-7 Early outcome: tubular function. Evolution of the fraction of excreted sodium in the urine in the first week post-transplant. Serial blood and urine samples were collected from transplanted pigs and processed for biochemical analysis. Shown are means±SD, n=7.
10.1186/s12967-016-1013-7 Immunofluorescent evaluation of complement deposition in kidney biopsies at 30min post-transplant: Factor B and C5b9.Needle biopsies were performed 30 min after unclamping (45 min post injection) and processed for cryopreservation. Slides were evaluated by fluorescence microscopy by a trained nephropathologist. **CTL**: Healthy kidney; Vehicle (**VEH**): injected with vehicle 15 min before unclamping; **rhC1INH**: Injected with 500 U/kg rhC1INH 15 min before unclamping (n=7). **A**: Representative staining for **Factor B**. **B**: Representative staining for **C5b9**. Magnification: X200.

## Abbreviations

AST: aspartate aminotransferase
CTL: control
DGF: delayed graft function
EMT: epithelial to mesenchymal transition
IRI: ischemia reperfusion injury
MASP: mannan-binding lectin serine protease 1
MBL: mannnose-binding lectin
Ngal: neutrophil gelatinase-associated lipocalin
rhC1INH: recombinant human C1 inhibitor
UFH: unfractionated heparin
UW: University of Wisconsin preservation solution
VEH: vehicle
