# Supplementary material for: Inhibition of complement improves graft outcome in a pig model of kidney autotransplantation
Source: J Transl Med. 2016 Sep 23;14:277. doi: 10.1186/s12967-016-1013-7 (PMC5035455; doi:10.1186/s12967-016-1013-7)
Supplement: Supplementary file 1 — 10.1186/s12967-016-1013-7 Power calculations for the different statistical analyses. Calculations were performed using Anastat (http://www.anastats.fr/) with an alpha threashold of 0.05. Table 2. Immune cell counts. [file 12967_2016_1013_MOESM1_ESM.docx]

**Supplementary Table 1: Power calculations for the different statistical analyses.**

Calculations were performed using Anastat (<http://www.anastats.fr/>) with an alpha threashold of 0.05.

|  |  | **Statistical Power of:** | |
| --- | --- | --- | --- |
| **Tested Parameter** | **Delta** | **Ttest** | **Mann-Whitney** |
| Creatininemia AUC | 5.88 | 100% | 95% |
| Creatininemia at M3 | 15.2 | 100% | 95% |
| Fibrosis scoring M3 | 7.82 | 100% | 95% |
| MAC387 Staining | 1.97 | 48% | 46% |
| CD3 Staining | 2.96 | 83% | 79% |
| Vimentin Staining M3 | 1.86 | 44% | 42% |
| aSMA staining M3 | 2.05 | 52% | 49% |
| TGFb Western Blot | 5.64 | 100% | 95% |
| Smad 3 western blot | 14.53 | 100% | 95% |

**Supplementary Table 2: Immune cell counts**

The number of cells was determined by counting the number of positive immune cells *per* field. We evaluated 10 fields (X100) *per* tissue sample.

|  | **VEH** | **rhC1INH** |
| --- | --- | --- |
| MAC-387 staining | 5.3±3.0 | 1.5±0.2 * |
| CD3 staining | 63.4±21.7 | 26.8±19.0 * |

Shown are means±SD, n=7. Statistics: *: p<0.05. MAC-387 data were normally distributed but did not show equality of variance on the Variance-Ratio Equal-Variance Test, hence non parametric analysis was performed. CD3 data was normally distributed and had equal variance, hence parametric analysis was performed.
