# Supplementary figures and images for: Inhibition of complement improves graft outcome in a pig model of kidney autotransplantation
Source: J Transl Med. 2016 Sep 23;14:277. doi: 10.1186/s12967-016-1013-7 (PMC5035455; doi:10.1186/s12967-016-1013-7)

## Slide 1
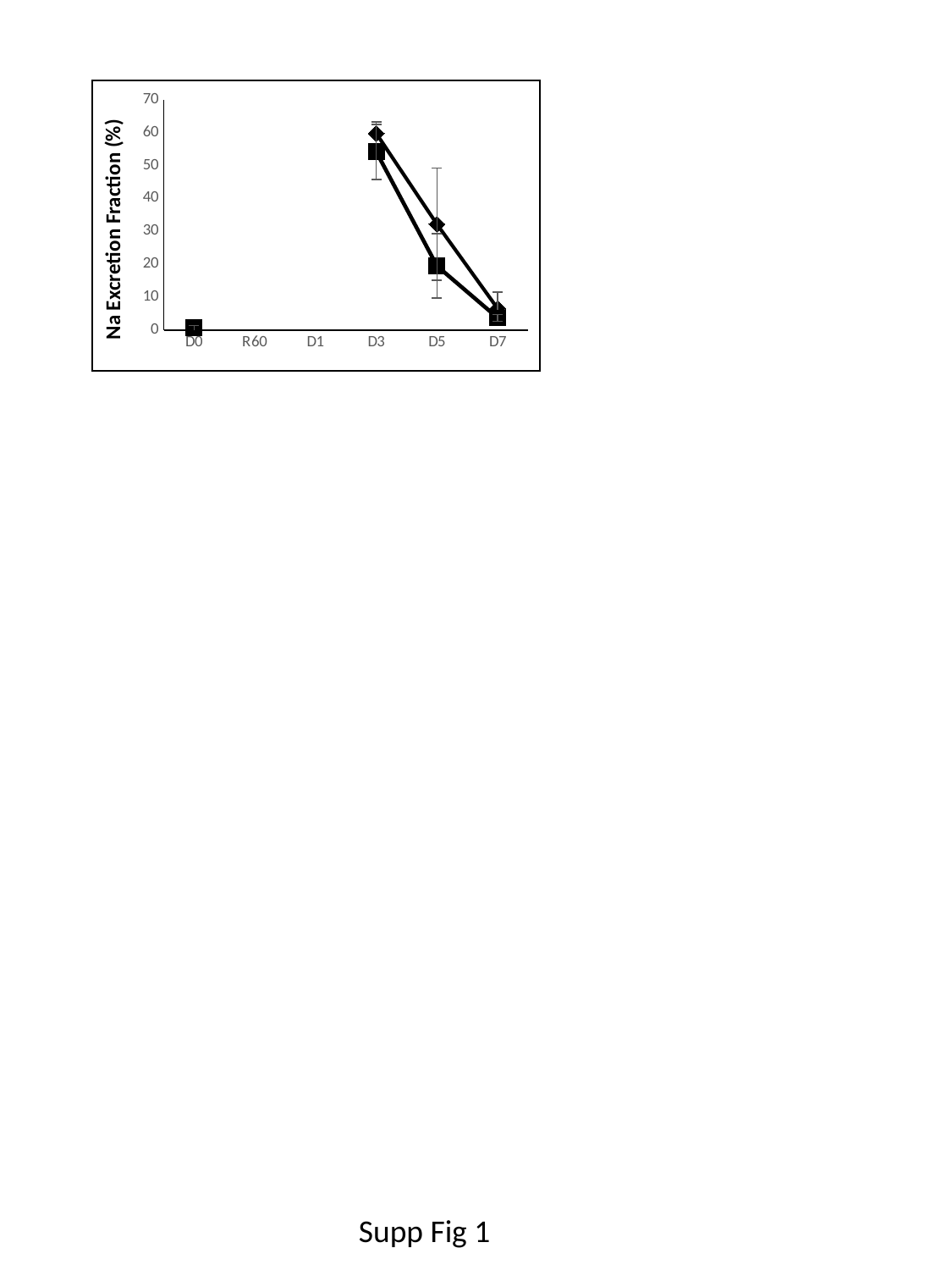

### Chart
| Category | Vehicle | rhC1INH |
|---|---|---|
| D0 | 0.8284044153023283 | 0.694709618785617 |
| R60 | None | None |
| D1 | None | None |
| D3 | 59.712040925728424 | 54.138099013773804 |
| D5 | 32.15242992779107 | 19.46282586304711 |
| D7 | 6.513233169787862 | 3.7015787964462774 |
Na Excretion Fraction (%)
Supp Fig 1

Supplement: Supplementary file 2 — 10.1186/s12967-016-1013-7 Early outcome: tubular function. Evolution of the fraction of excreted sodium in the urine in the first week post-transplant. Serial blood and urine samples were collected from transplanted pigs and processed for biochemical analysis. Shown are means±SD, n=7. [file 12967_2016_1013_MOESM2_ESM.pptx]

## Slide 1
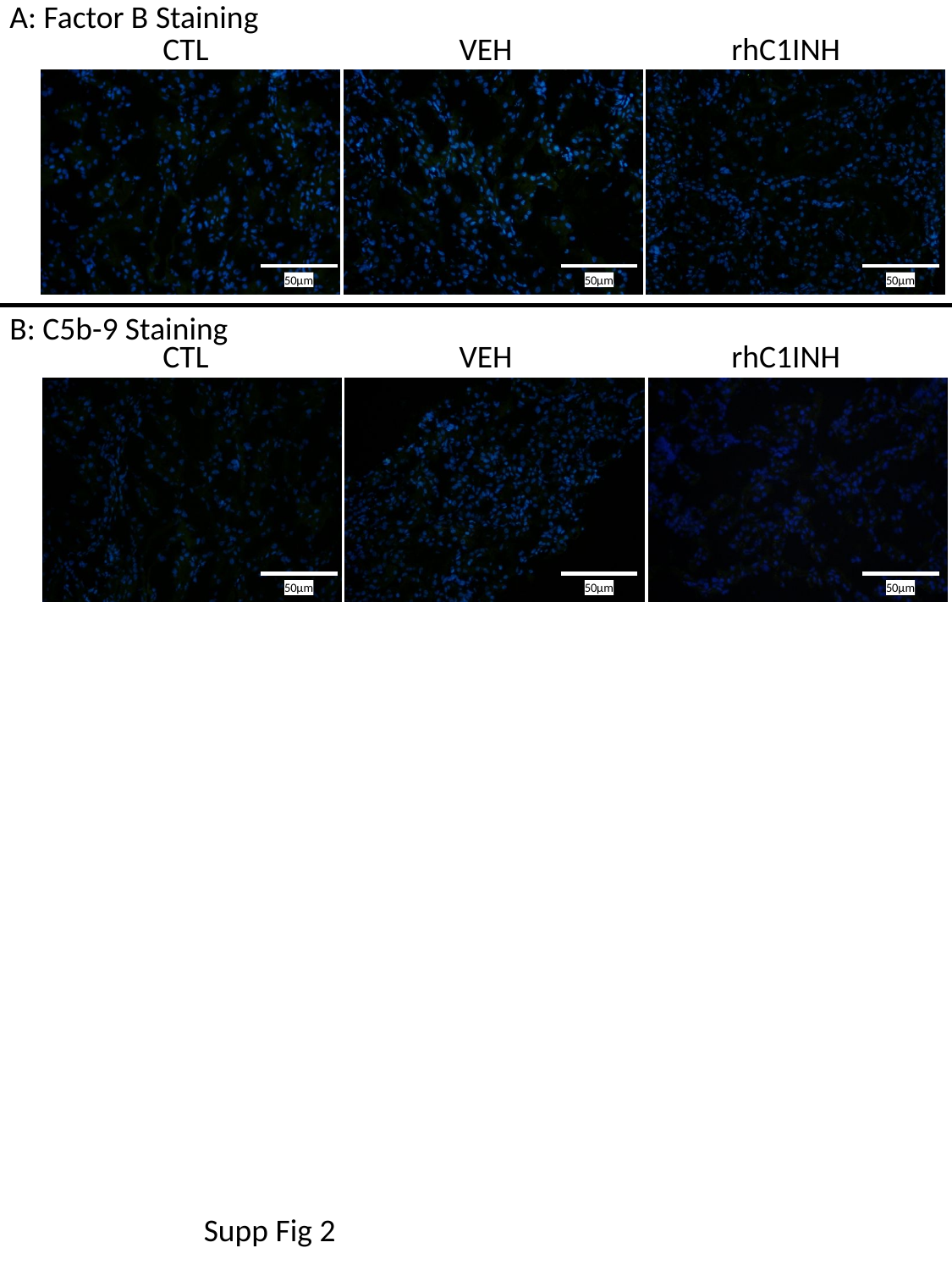

A: Factor B Staining
CTL
VEH
rhC1INH
50µm
50µm
50µm
B: C5b-9 Staining
CTL
VEH
rhC1INH
50µm
50µm
50µm
Supp Fig 2

Supplement: Supplementary file 3 — 10.1186/s12967-016-1013-7 Immunofluorescent evaluation of complement deposition in kidney biopsies at 30min post-transplant: Factor B and C5b9.Needle biopsies were performed 30 min after unclamping (45 min post injection) and processed for cryopreservation. Slides were evaluated by fluorescence microscopy by a trained nephropathologist. CTL: Healthy kidney; Vehicle (VEH): injected with vehicle 15 min before unclamping; rhC1INH: Injected with 500 U/kg rhC1INH 15 min before unclamping (n=7). A: Representative staining for Factor B. B: Representative staining for C5b9. Magnification: X200. [file 12967_2016_1013_MOESM3_ESM.pptx]
